# Supplementary material for: Chlorinated Electron Acceptor Abundance Drives Selection of Dehalococcoides mccartyi (D. mccartyi) Strains in Dechlorinating Enrichment Cultures and Groundwater Environments
Source: Front Microbiol. 2018 May 17;9:812. doi: 10.3389/fmicb.2018.00812 (PMC5968391; doi:10.3389/fmicb.2018.00812)
Supplement: Supplementary file 1 [file Data_Sheet_1.pdf]

SUPPORTING INFORMATION

Chlorinated Electron Acceptor Abundance Drives Selection of *Dehalococcoides mccartyi (D. mccartyi)* Strains in Dechlorinating Enrichment Cultures and Groundwater Environments

A. Pérez-de-Mora[1,2] A. Lacourt[1], M.L. McMaster[3], X. Liang[1], S.M. Dworatzek[4], E.A. Edwards[1]

[1] Dept. of Chemical Engineering and Applied Chemistry, University of Toronto, 200 College St., Toronto, ON M5S 3E5N, Canada

[2] Research Unit Analytical Biogeochemistry, Dept. of Environmental Sciences, Helmholtz Zentrum München, Ingolstädterlandstr. 1, 85764 Neuherberg, Germany

[3]Geosyntec Consultants, Guelph, ON, Canada

[4]SiREM, Guelph, ON, Canada

TABLE OF CONTENTS

Table S1. Summary of cultures investigated -- Page 3

Table S2. Summary of *rdhA* genes in the mixed dechlorinating culture KB-1 ----- Page 4

Table S3. List of *rdhA* targets (and OG numbers) and primer pairs for qPCR ----- Page 5

Table S4. Primer – Analysis of sequence mismatches -------------------------------- Page 6

Table S5. qPCR standard curves --- Page 7

Table S6. qPCR data for Figure 2a (U of T cultures) of the main document ------- Page 8

Table S7. qPCR data for Figure 2b (Sirem Cultures) of the main document ------- Page 9

Table S8. qPCR data for Figure 3 (field sites) of the main document -------------- Page 10

Table S9. Detection limits for all qPCR data --- Page 11

Figure S1. Sketch of the ISSO site in Canada -- Page 12

Figure S2. Sketch of the Sabre site in the UK -- Page 13

Figure S3. Phylogenetic tree based on the nucleotide sequences of the *rdhA* genes of *Dehaloccocoides* strains and the mixed dechlorinating culture KB-1 ------------ Page 14

References --- Page 15

Table S1. Summary of cultures investigated for the presence of both *Dehalococcoides* and *rdh*A genes.

**Culture/KB1 enrichment**	**Main dechlorinating organism***	**Sampling dates**	**e- donor**	**e- acceptor**	**Main reaction**	**Feeding**	**Target conc. Of electron acceptor/ Vol. liquid/ Vol. headspace**
TCE/M_1998_Parent	*Dhc* & *Geo*	11-Jan-11	M	TCE	TCE to ETH	400 µL 5:1 stock (M/TCE)	0.68 mmol L^{-1} 1.6L/0.4L
TCE/M_1999	*Dhc* & *Geo*	25-Oct-11	M	TCE	TCE to ETH	400 µL 5:1 stock (M/TCE)	0.68 mmol L^{-1} 1.6L/0.4L
TCE/M_2010	*Dhc* & *Geo*	spring 2011	M	TCE	TCE to ETH	30 µL 5:1 stock (M/TCE)	0.68 mmol L^{-1} 0.2L/0.0.05L
TCE/ME_2001_SiREM	*Dhc* & *Geo*	2004, 05, 06, 07 and 09	ME	TCE	TCE to ETH	4 mL 5:1 (M/TCE) 1mL E	0.15-0.19 mmol L^{-1} 99L/1L
TCE/H2_2001	*Dhc* & *Geo*	11-Jan-11/ 25-Oct-11	H_2	TCE	TCE to ETH	60 mL- 100 mL H_2/15µL TCE	0.19 mmol L^{-1} 0.8L/0.2L
cDCE/M_2001	*Dhc*	7-Jun-11/ 25-Oct-11	M	cDCE	cDCE to ETH	110µL 5:1 stock (M/cDCE)	0.46 mmol L^{-1} 1.7L/0.3L
cDCE/M_2003	*Dhc*	07-Jun-11	M	cDCE	cDCE to ETH	50 µL 5:1 stock (M/cDCE)	0.46 mmol L^{-1}/ 0.65L/0.35L
VC/M_2001	*Dhc*	25-Oct-11	M	VC	VC to ETH	53 µL/20 mL VC	0.69 mmol L^{-1} 1L/0.2L
VC/H2_2003-1	*Dhc*	11-Jan-11	H_2	VC	VC to ETH	20 mL H_2/4 mL VC	0.72 mmol L^{-1} 0.2L/0.25L
VC/H2_2003-2	*Dhc*	11-Jan-11/ 25-Oct-11	H_2	VC	VC to ETH	5 mL H_2/1 mL VC	0.16 mmol L^{-1}/ 0.2L/0.05L
VC/M_2004	*Dhc*	25-Oct-11	M	VC	VC to ETH	5 µL/2 mL VC	0.69 mmol L^{-1}/ 0.12L/0.06L
12DCA/M_2008_UT	*Dhc*	11-Jan-11/ 25-Oct-11	M	1,2-DCA	1,2-DCA to ETH	46 µL 5:1 stock (M/1,2-DCA)	0.2 mmol L^{-1} 1.5L/0.5L
12DCA/ME_2010_SiREM	*Dhc*	7-Jun-11/ 25-Oct-11	ME	1,2-DCA	1,2-DCA to ETH	50 µL 5:5:1 stock (M/E/1,2-DCA)	1.26 mmol L^{-1}/ 0.2L /0.05L

cDCE = cis-dichloroethene; DCA = dichloroethane; *Dhc* = *Dehalococcoides*; E = ethanol; ETH = Ethene; *Geo* = *Geobacter*; M = methanol; ME = methanol/ethanol; TCE = trichloroethene; VC = vinyl chloride. *based on PCR, clone libraries and metagenome analysis. The name format for the enrichment cultures indicates electron acceptor amended/donor used_year created (e.g. VC/M_2001 is a VC and methanol enrichment culture first established in 2001 and VC/H2_2003-2 is a second enrichment culture established in 2003 with VC and hydrogen). Cultures in grey were not used in this study, but are provided for completeness, as they are available for research and have been discussed in other publications.

Table S2. Compiled reductive dehalogenase sequences identified in the dechlorinating enrichment culture KB-1.

Name in paper	OG**	Previous name	Name in Hug et al., 2013[1]	Accession number	NCBI GI	JGI locust Tag	JGI/IMG name	metagenome contig ID
KB1_1	6	KB1_1	KB1_1 & KB13109_4*	DQ177506	77176847	DCKB1_110270	none	none
KB1_2	11	KB1_2	KB1_2	DQ177507	77176850	DCKB1_110450	2013897470	C3109
KB1_3	12	KB1_3	KB1_3	DQ177508	77176853	DCKB1_11560	2013887593	C299
KB1_4	13	KB1_4	KB1_4	DQ177509	77176856	DCKB1_110180	2013897443	C3109
KB1_5	15	KB1_5 (DET 1545)	KB1_5	DQ177510	77176859	DCKB1_110110	2013897436	C3109
KB1_6 (*bvcA*)	28	KB1_6 (*bvcA*)	KB1_6	DQ177511	77176862	none	none	FOFA22070
KB1_7	19	KB1_7	KB1_7 & KB13241_7*	DQ177512	77176865	DCKB1_114830	none	none
KB1_8	17	KB1_8	KB1_8	DQ177513	77176868	DCKB1_11540	2013887591	C299
KB1_9	17	KB1_9	KB1_9	DQ177514	77176871	DCKB1_110210	2013897446	C3109
KB1_10	34	KB1_10	KB1_10 & KB13109_9*	DQ177515	77176874	DCKB1_110480	2013897473	C3109
KB1_11	37	KB1_11	KB1_11 & KB13240_1*	DQ177516	77176877	DCKB1_114790	none	C3240
KB1_12	18	KB1_12	KB1_12	DQ177517	77176880	DCKB1_110630	2013897488	C3109
KB1_13 (*pceA*)	30	KB1_13 (*pceA*)	KB1_13	DQ177518	77176883	DCKB1_110540	2013897479	C3109
KB1_14 (*vcrA*)	8	KB1_14 (*vcrA*)	KB1_14	DQ177519	77176886	DCKB1_96900	2013896112	C2841
KB1_15	39	C3241_1	KB13241_1	KP085015	733372930	DCKB1_115000	2013897921	C3241
KB1_16	50	C3241_2	KB13241_2	KP085016	733372933	DCKB1_115150	2013897936	C3241
KB1_17	49	C3241_3	KB13241_3	KP085017	733372936	DCKB1_115210	2013897942	C3241
KB1_18	14	C3241_4	KB13241_4	KP085018	733372939	DCKB1_115020	2013897923	C3241
KB1_19	36	C3241_5	KB13241_5	KP085019	733372942	DCKB1_115090	2013897930	C3241
KB1_20	38	C3241_6	KB13241_6	KP085020	733372945	DCKB1_114910	2013897912	C3241
KB1_21	32	C3241_8	KB13241_8	KP085021	733372948	DCKB1_114860	2013897907	C3241
KB1_22	40	KB1_22	KB1_22 & KB13109_7*	JX081249	393716494#	DCKB1_110600	none	none
KB1_23	29	C3107_1	KB13107_1	KP085022	733372951	DCKB1_107470	2013897169	C3107
KB1_24	22	C3107_2	KB13107_2	KP085023	733372954	DCKB1_107520	2013897174	C3107
KB1_25	56	C1024_1	KB11024_1	KP085024	733372957	DCKB1_37290	2013890159	C1024
KB1_26	23	C3108_1 (cons.synt)	KB13108_1	KP085025	733372960	DCKB1_107910	2013897212	C3108
KB1_27 (*tceA*)	5	C338_1 (*tceA*)	KB1338_1	KP085026	733372963	DCKB1_14890	2013887924	C338
KB1_28	26	KB1_group26	n/a	KP085027	733372966	DCKB1_13760	2013887811	C338
KB1_29 (partial)	n/a	KB1_F43012	n/a	KP085028	733372969	DCKB1_297220	2013916109	FOFA43012
KB1_30 (partial)	n/a	KB1_F51719	n/a	KP085029	733372971	DCKB1_319470	2013918333	FOFA51719
Geo_rdh	41	KB-1_Geo_rdase	n/a	JX081248	393716492#	DCKB1_87010; DCKB1_86980	none	Geo_RD

*possibly two highly similar sequences (>99%) from different sequencing efforts as presented in reference (1) - Hug *et al.*, 2013.

**OG: ortholog group number, as defined in reference (1)

cons.synt. = conserved syntenic; OG = Ortholog Group

#accession numbers reported in reference (2) - Tang *et al.*, 2013. Note that accession number for Geo_RD in (2) was listed erroneously as 393716494, instead of 393716492.

Table S3. List of rdhA targets with corresponding OG and primer pairs for qPCR employed in this study. Unless specified otherwise, primers were specifically designed for this study.

**OTU/functional gene** *(accession #)* **Ortholog Group Number**	**Primer pair**	**sequence**	**Annealing (Celsius)**	**fragment length (bp)**
Dehalococcoides 16S rRNA (AY146779.1)	Dhc 1f(3)<br>Dhc 264r(3)	5'-GATGAACGCTAGCGGCG-3'<br>5'-CCTCTCAGACCAGCTACCGATCGAA- 3'	60	264
KB1_1 (DQ177506)<br>**OG10**	KB1_1_246f(4)<br>KB1_1_336r(4)	5'-ATCGGAGCTGCACAAGTAGG- 3'<br>5'-TCTTGTGAGCGGTGTCTTTG- 3'	60	91
KB1_4 (DQ177509)<br>**OG13**	KB1_4_310f<br>KB1_4_642r	5'-GCTCTTAACGCAGGGGCAA- 3'<br>5'-GCGGCTGGCATCTACAGG- 3'	63	333
KB1_5 (DQ177510)<br>**OG15**	KB1_5_1017f(4)<br>KB1_5_1137r(4)	5'-GATGCAGGCATTTACCGTTT- 3'<br>5'-GTCTCTTTGCCTTCGGTCAG- 3'	60	121
KB1_6 (*bvcA*) (DQ177511)<br>**OG28**	KB1_6 _318f(4)<br>KB1_6 _555r(4)	5'-ATTTAGCGTGGGCAAAACAG- 3'<br>5'-CCTTCCCACCTTGGGTAT TT- 3'	60	238
KB1_11 (DQ177516)<br>**OG37**	KB1_11_723f<br>KB1_11_943r	5'-CCACCAATATCACCATACCTAA- 3'<br>5'-ACCGGTGGGAATGAAACG- 3'	59	221
KB1_12 (DQ177517)<br>**OG18**	KB1_12_804f<br>KB1_12_1041r(4)	5'-TTCATCACGACAGCTTGGTG- 3'<br>5'-TTTGGGGGTCATAACTGCTC- 3'	60	238
KB1_14 (*vcrA*) (DQ177519)<br>**OG8**	KB1_14 _642f(4)<br>KB1_14 _846r(4)	5'-GAAAGCTCAGCCGATGACTC- 3'<br>5'-TGGTTGAGGTAGGGTGAAGG- 3'	60	205
KB1_15 (KP085015)<br>**OG39**	KB1_15_491f<br>KB1_15_847r	5'-GTCCCGAAGAGACTACCGCT- 3'<br>5'-TGCCAAGACCCTGATAGCCT- 3'	66	357
KB1_16 (KP085016)<br>**OG50**	KB1_16 _727f<br>KB1_16 _955r	5'-GCCAGCCCGATACGTGAACTG- 3'<br>5'-GGAACCGGCGCTGAACTATGG- 3'	65	229
KB1_17 (KP085017)<br>**OG49**	KB1_17_259f<br>KB1_17_590r	5'-GTCCTTATAGAATACGCCCAGA- 3'<br>5'-GTACCAATACAAGTCTCTCCAG- 3'	63	332
KB1_18 (KP085018)<br>**OG14**	KB1_18_414f<br>KB1_18_556r	5'-GGCTTATCCTGGTATCGTA- 3'<br>5'-AACCGATAAACATGGCAC- 3'	57.5	143
KB1_19 (KP085019)<br>**OG36**	KB1_19_1086f<br>KB1_19_1283r	5'-CCTAACCAACCGGGCAAT- 3'<br>5'-CGCCAGCCGAGAAAACC- 3'	54	198
KB1_23 (KP085022)<br>**OG29**	KB1_23_431f<br>KB1_23_636r	5'-TTGGTCAGGGTCGGTCA- 3'<br>5'-TTCACCAACATTCAGGACTC- 3'	57	206
KB1_25 (KP085024)<br>**OG56**	KB1_25_397f<br>KB1_25_613r	5'-TGCGAAATGGGCGGGATG- 3'<br>5'-GCACTGAAGGCCCGAATGTAC- 3'	60	216
KB1_27 (*tceA*) (KP085026)<br>**OG5**	tceA500f(5)<br>tceA795r(5)	5'-TAATATATGCCGCCACGAATGG- 3'<br>5'-ATCGTATACCAAGGCCCGAGG- 3'	64	296
Geobacter 16S rRNA	Geo_73f(3)<br>Geo_485r(3)	5'-CTTGCTCTTTCATTTAGTGG- 3'<br>5'-AAGAAAACCGGGTATTAA CC- 3'	66	413
Geobacter_rdhA (JX081248)	GeordhA_812f<br>GeordhA_1045r	5'-AGCTGAGTCAGGGTTTGAGCC- 3'<br>5'-CCATACGACTGCCTTCACCCAT- 3'	66	234

References: Duhamel and Edwards 2006: Waller *et al.* 2005; Fung *et al.* 2007;   OG – ortholog group

Table S4. Mismatches between primers and sequences in Ortholog Groups (OG). Note that primers were designed to target KB1 sequences and not the OG groups.

OTU/functional gene	Primer pair	primer specificity to OG group<br>number of primer-sequence mismatches
KB1_1<br>OG10 (7 sequences)	KB1_1_246f[(4)]<br>KB1_1_336r[(4)]	4 mismatches – VS_1421, GY50_1427; 1 mismatch (rest of sequences)<br>3 mismatches – AY374245, btf_1481; 5 mismatches VS_1421, GY50_1427
KB1_4<br>OG13 (9 sequences)	KB1_4_310f<br>KB1_4_642r	4 mismatches – VS_1430, GY50_1436<br>1 mismatches – VS_1430, GY50_1436
KB1_5<br>OG15 (10 sequences)	KB1_5_1017f[(4)]<br>KB1_5_1137r[(4)]	4 mismatches – DET_1545<br>1 mismatches – DET_1545, 2 mismatches – btf_1497, GY50_1443
KB1_6 (*bvcA*)<br>OG28 (2 sequences)	KB1_6 _318f[(4)]<br>KB1_6 _555r[(4)]	0 mismatches<br>0 mismatches
KB1_11<br>OG37 (3 sequences)	KB1_11_723f<br>KB1_11_943r	0 mismatches<br>0 mismatches
KB1_12<br>OG18 (3 sequences)	KB1_12_804f<br>KB1_12_1041r[(4)]	5 mismatches GY50_1408<br>3 mismatches GY50_1408
KB1_14 (*vcrA*)<br>OG8 (4 sequences)	KB1_14 _642f[(4)]<br>KB1_14 _846r[(4)]	0 mismatches<br>1 mismatches all four sequences
KB1_15<br>OG39 (5 sequences)	KB1_15_491f<br>KB1_15_847r	0 mismatches<br>0 mismatches
KB1_16<br>OG50 (3 sequences)	KB1_16 _727f<br>KB1_16 _955r	0 mismatches<br>0 mismatches
KB1_17<br>OG49 (3 sequences)	KB1_17_259f<br>KB1_17_590r	0 mismatches<br>0 mismatches
KB1_18<br>OG14 (6 sequences)	KB1_18_414f<br>KB1_18_556r	0 mismatches<br>0 mismatches
KB1_19<br>OG36 (3 sequences)	KB1_19_1086f<br>KB1_19_1283r	0 mismatches<br>0 mismatches
KB1_23<br>OG29 (4 sequences)	KB1_23_431f<br>KB1_23_636r	1 mismatch – DET_0235; 3 mismatches – GY50 0087<br>4 mismatches – GY50_0087; 7 mismatches - DET 0235
KB1_25<br>OG56 (2 sequences)	KB1_25_397f<br>KB1_25_613r	0 mismatches<br>0 mismatches
KB1_27 (*tceA*)<br>OG5 (3 sequences)	tceA500f[(5)]<br>tceA795r[(5)]	0 mismatches<br>0 mismatches
Geobacter_rdhA	GeordhA_812f<br>GeordhA_1045r	0 mismatches<br>0 mismatches

References: Waller *et al.* 2005; Fung *et al.* 2007; OG – ortholog group

Table S5. Details of the standard curves generated for qPCR with the different primer pairs, including average slopes, Y-intercepts, R^2 and their corresponding standard deviations.

**Target**	**m**		**n**		**R**		**Eff**		**N**
	**average**	**stdev**	**average**	**stdev**	**average**	**stdev**	**average**	**stdev**	
Dhc 16S	-3.462	0.030	35.179	0.596	0.998	0.002	94.5	1.1	4
**KB1_1**	-3.225	NA	34.180	NA	0.997	NA	104	NA	1
**KB1_4**	-3.479	0.201	34.930	1.39	0.999	0.000	94.2	7.8	3
**KB1_5**	-3.515	NA	35.063	NA	0.999	NA	92.5	NA	1
**KB1_6 *(bvcA)***	-3.488	0.016	35.520	0.373	0.9972	0.002	93.5	0.6	2*
**KB1_11**	-3.427	0.082	35.215	0.596	0.999	0.000	95.8	3.1	3
**KB1_12**	-3.350	0.322	36.177	0.327	0.996	0.006	99.2	14	2*
**KB1_14 (*vcrA*)**	-3.381	0.142	35.280	1.727	0.998	0.001	97.7	5.7	2*
**KB1_15**	-3.328	0.136	35.231	0.386	0.994	0.004	99.8	5.6	2*
**KB1_16**	-3.353	0.126	35.405	1.000	0.998	0.001	96.2	4.7	3
**KB1_17**	-3.464	0.001	35.759	0	0.997	0.000	94.4	0.0	2*
**KB1_18**	-3.333	0.043	34.086	1.829	0.998	0.002	99.5	1.8	2*
**KB1_19**	-3.174	0.001	34.069	0.02	0.998	0.000	107	0.1	2*
**KB1_23**	-3.442	0.094	34.936	0.109	0.997	0.001	95.3	3.5	3
**KB1_25**	-3.473	0.167	35.540	0.163	0.997	0.000	94.2	6.2	2*
**KB1_27 *(tceA)***	-3.512	NA	35.087	NA	0.996	NA	92.6	NA	1
Geobacter 16S	-3.520	NA	35.579	NA	0.994	NA	92.3	NA	1
Geobacter_rdhA	-3.394	0.2382	35.161	0.784	0.997	0.0029	99.4	9.8	2*

avg = average; *Dhc* = *Dehalococcoides*. Eff = efficiency; m = slope; n = Y-intercept; R^2 = coefficient of determination; N = number of standard curves used for the calculations; NA = not applicable; stdev = standard deviation.

* Note that when N = 2 the range, that is, the difference between the two values available was calculated instead of the standard deviation.

Table S6. Absolute qPCR gene copy numbers per L of culture for each *rdhA* gene used for the calculation of the *rdhA*/*Dhc* ratios of Figure 2a of the main manuscript. Note that values for the 16S rRNA of *Geobacter* strain KB-1 and the *Geobacter rdhA* gene for some of the cultures are also included, although they do not appear on Figure 2.

	**TCE/M 1999 jan11**	**TCE/H2 2001 jan11**	**TCE/ME 2001 SiREM oct09**	**TCE/M 1998 parent oct11**	**TCE/M 2010 mar11**	**VC/H2 2003-1 jan11**	**VC/H2 2003-2 jan11**	**VC/H2 2003-2 oct11**	**cDCE/M 2003 jun11**	**cDCE/M 2001 jun11**	**cDCE/M 2001 oct11**	**1,2DCA/ M 2008 UToct11**	**1,2DCA/ M 2008 UTjan11**	**1,2DCA/ ME 2010 SiREM**
Dhc 16S	1.7E+8	1.5E+9	7.1E+8	4.4E+10	2.3E+10	3.9E+9	1.3E+9	1.3E+10	1.2E+9	1.3E+9	6.9E+9	1.2E+10	5.5E+8	2.2E+9
**KB1_25**	1.4E+6	1.4E+6	≤8.2E+5	1.6E+9	5.4E+8	≤8.2E+5	≤8.2E+5	≤8.2E+5	≤8.2E+5	≤8.2E+5	≤8.2E+5	2.2E+10	3.5E+8	1.0E+9
**KB1_16**	1.9E+6	1.8E+6	≤8.7E+5	1.0E+9	6.6E+8	≤8.7E+5	≤8.7E+5	≤8.7E+5	≤8.7E+5	≤8.7E+5	≤8.7E+5	1.4E+10	3.8E+8	1.2E+9
**KB1_17**	≤5.0E+5	5.0E+5	≤5.0E+5	3.4E+8	2.8E+8	≤5.0E+5	≤5.0E+5	≤5.0E+5	≤5.0E+5	≤5.0E+5	≤5.0E+5	3.9E+9	1.5E+8	5.3E+8
**KB1_19**	2.6E+6	2.5E+6	9.1E+5	1.7E+9	6.4E+8	9.8E+6	3.2E+6	2.3E+07	≤8.3E+5	≤8.3E+5	≤8.3E+5	1.9E+10	5.7E+8	1.2E+9
**KB1_27**	3.5E+7	3.9E+7	3.7E+8	1.8E+10	1.0E+6	2.5E+6	8.5E+5	NA	7.0E+5	5.3E+5	NA	NA	4.1E+8	1.1E+9
**KB1_6**	1.1E+6	3.0E+6	≤1.0 E+6	≤1.0E+6	3.9E+6	≤1.0 E+6	≤1.0 E+6	≤1.0 E+6	5.5E+8	4.1E+8	4.1E+9	1.2E+9	2.1E+7	5.3E+8
**KB1_15**	3.5E+6	2.7E+6	8.9E+5	1.0E+9	1.2E+9	1.3E+10	2.5E+9	6.0E+9	3.8E+8	5.9E+8	2.4E+9	1.3E+10	7.4E+8	2.2E+9
**KB1_18**	3.7E+6	1.3E+6	≤1.0E+6	1.2E+9	7.5E+8	6.5E+9	2.4E+9	4.6E+9	1.4E+8	3.0E+8	1.3E+9	1.5E+10	6.7E+8	1.2E+9
**KB1_5***	4.1E+8	1.6E+9	7.9E+8	5.0E+10	1.6E+9	1.0E+10	2.9E+9	NA	6.6E+8	1.3E+8	NA	NA	3.4E+8	1.3E+9
**KB1_23**	3.1E+8	9.4E+8	5.3E+8	3.5E+10	2.2E+10	2.5E+9	5.0E+8	8.6E+9	3.5E+7	5.1E+5	2.9E+6	4.9E+8	9.9E+6	2.0E+8
**KB1_12**	4.2E+7	6.0E+8	2.2E+9	5.4E+10	6.0E+9	6.3E+8	6.8E+7	1.4E+10	2.2E+8	4.3E+5	9.1E+6	1.8E+9	3.6E+6	6.5E+7
**KB1_4**	3.1E+8	8.6E+8	5.0E+8	5.0E+10	NA	8.3E+9	3.1E+9	1.4E+10	4.1E+8	2.9E+8	1.3E+9	1.0E+10	NA	NA
**KB1_11**	4.6E+7	7.2E+8	7.1E+7	1.8E+9	1.2E+9	1.2E+10	5.1E+9	6.3E+09	2.5E+8	5.0E+8	1.8E+9	2.7E+8	1.2E+7	1.3E+7
**KB1_14**	7.9E+7	5.8E+8	7.1E+8	4.7E+10	8.7E+9	7.1E+9	9.7E+8	2.8E+10	7.4E+8	7.4E+8	8.0E+9	7.1E+9	7.9E+7	6.2E+8
**KB1_1***	5.7E+8	3.2E+9	1.0E+9	1.1E+11	2.1E+9	1.9E+10	5.4E+9	NA	2.8E+8	4.8E+8	8.9E+9	NA	5.2E+8	2.4E+9
Dhc 16S*	4.2E+8	2.5E+9	1.5E+9	1.1E+11	2.2E+9	2.1E+10	4.7E+9	NA	2.7E+8	4.9E+8	1.3E+9	NA	4.4E+8	2.2E+9
Geo 16S	NA	NA	1.6E+8	5.9E+9	NA	NA	≤1.1E+6	≤1.1E+6	NA	NA	≤1.1E+6	≤1.1E+6	NA	NA
Geo_rdh	3.3E+7	1.0E+9	1.5E+8	7.8E+9	2.0E+9	≤3.6E+5	≤4.6E+5	≤4.6E+5	≤4.6E+5	≤4.6E+5	≤4.6E+5	≤4.6E+5	≤4.6E+5	≤4.6E+5

*Absolute quantification for KB1_1, KB1_5 and KB1_27 was conducted on a different date than for the rest of *rdhA* genes and thus values for these genes were referred to *Dhc* values of the corresponding date. *Dhc* = *Dehalococcoides*; NA = not analyzed; *Geo* = *Geobacter*; ≤ MDL value is equal to or lower than the method detection limit. KB1 27 (*tceA*); KB1_6 (*bvcA*); KB1_14(*vcrA*).

Note: The difference between duplicate measurements was usually around 5% or less of absolute value of each duplicate measurement.

Table S7. Absolute qPCR gene copy numbers per L of culture for each *rdhA* used for Figure 2b of the main manuscript.

	**TCE/ME 2001 Sir Feb 2004**	**TCE/ME 2001 Sir Jan 2005**	**TCE/ME 2001 Sir Mar 2006**	**TCE/ME 2001 Sir Apr 2007 SaBRE inoculum**	**TCE/ME 2001 Sir Oct 2009 ISSO inoculum**
Dhc 16S	6.8E+10	2.0E+11	5.3E+10	3.5E+10	7.1E+08
**KB1_25**	≤8.2E+05	≤8.2E+05	≤8.2E+05	≤8.2E+05	≤8.2E+05
**KB1_16**	≤8.7E+05	≤8.7E+05	≤8.7E+05	≤8.7E+05	≤8.7E+05
**KB1_17**	≤5.0E+05	≤5.0E+05	≤5.0E+05	≤5.0E+05	≤5.0E+05
**KB1_19**	8.9E+07	1.5E+08	1.2E+08	1.3E+08	9.1E+05
**KB1_6/*bvcA***	7.0E+07	8.5E+09	2.3E+09	3.2E+08	≤1.0 E+06
**KB1_15**	4.5E+10	1.9E+10	6.7E+08	7.6E+08	8.9E+05
**KB1_18**	7.9E+10	2.0E+10	3.9E+08	6.3E+08	≤1.0E+06
**KB1_23**	6.0E+10	2.6E+11	4.7E+10	3.7E+10	5.3E+08
**KB1_12**	6.8E+10	3.6E+11	7.2E+10	5.6E+10	2.2E+09
**KB1_4**	6.0E+10	2.1E+11	NA	4.0E+10	5.0E+08
**KB1_11**	9.6E+10	1.3E+11	1.5E+10	2.6E+10	7.1E+07
**KB1_14/*vcrA***	2.7E+11	6.1E+11	9.7E+10	7.5E+10	7.1E+08
Geobacter 16S	4.3E+09	3.3E+10	5.0E+09	4.6E+09	1.6E+08
Geo_rdhA	2.1E+10	7.8E+10	6.6E+09	3.9E+09	1.5E+08

Dhc = 16S rRNA gene of *Dehalococcoides*; *Geo_rdhA* = *Geobacter rdhA*; *Geobacter* = 16S rRNA gene of *Geobacter*; NA = not analyzed. ≤ MDL value is equal to or lower than the method detection limit. Note: The difference between duplicate measurements was usually around 5% or less of the absolute value of each duplicate measurement.

Table S8. Absolute qPCR gene copy numbers per L of groundwater or per L of culture (for KB-1 inoculum) for each *rdhA* gene from which *rdhA*/*Dhc* ratios for Figure 4 of the main manuscript were calculated.

**site**	ISSO[1]	ISSO[1]	ISSO[1]	ISSO[1]	ISSO[1]	ISSO[1,2]	SABRE[1]	SABRE[1]	SABRE[1]	SABRE[1]	SABRE[1]	SABRE[1,3]	SABRE[1,3]
sample	**prebiost month 1**	**pre-bioaug month 18**	**KB-1 inoc.**	**post-bioaug month 22**	**postbioaug month 30**	**SW70 month 0**	**KB-1 inoc.**	**SW70 month 2**	**SW70 month 10**	**SW70 month 15**	**SW75 month 15**	**effluent month 15**	**influent month 15**
**Reme-diation status**	2.5 mos. before start of biostim.	15 mos. of biostim. and 1 mo. before bioaug.		19 mos. of biostim. and 3 mos. after bioaug..	27 mos. of biostim. and 11 mos. after bioaug..	prior to biostim. and bioaug.		2 weeks after bioaug. and 1 mo. after	8.5 mos. after bioaug. and 9 mos. after biostim.	13.5 mos. after bioaug. and 14 mos. after biostim.	13.5mos. after bioaug. and 14 mos. after biostim.	13.5 mos. after bioaug. and 14 mos. after biostim.	13.5 mos. after bioaug. and 14 mos. after biostim.
Dhc 16S	1.1E+6	1.5E+6	7.1E+8	1.2E+6	5.9E+6	≤6.2E+5	3.5E+10	1.4E+8	2.6 E+6	5.3 E+7	6.4 E+6	3.4 E+7	≤6.2E+5
**KB1_4**	3.8E+5	7.4E+5	5.0E+8	7.3E+5	2.5E+6	≤8.3E+5	4.0E+10	3.1E+8	4.4E+6	4.6E+7	8.4E+6	5.0E+7	≤8.3E+5
bvcA	4.4E+5	5.0E+5	≤1.0 E+6	1.1E+5	1.6E+6	≤3.4E+5	3.2E+8	≤3.4E+5	2.1E+6	1.1E+8	2.1E+7	6.5E+7	≤3.4E+5
**KB1_11**	≤2.4 E+5	7.7E+5	7.1E+7	3.6E+5	2.9E+6	≤9.6E+5	2.6E+10	9.2E+7	9.7E+5	6.4E+6	≤9.7E+5	≤9.7E+5	≤9.7E+5
**KB1_12**	≤1.8 E+5	9.0E+4	2.2E+9	1.3E+5	5.1E+4	≤4.6E+5	5.6E+10	1.2E+8	≤4.6E+5	5.9E+6	≤4.6E+5	1.8E+6	≤4.6E+5
vcrA	≤6.2 E+4	1.4E+6	7.1E+8	7.9E+5	1.1E+6	≤4.2E+5	7.5E+10	3.2E+8	2.E+6	4.4E+7	≤8.1E+3	1.1E+7	≤8.1E+3
**KB1_15**	≤6.0 E+4	7.7E+5	8.9E+05	3.5E+5	1.9E+6	≤2.8E+4	7.6E+8	6.5E+4	≤2.8E+4	2.1E+6	≤2.8E+4	8.8E+5	≤2.8E+4
**KB1_16**	≤1.8 E+5	1.3E+5	≤8.7E+5	7.1E+4	3.8E+5	≤5.8E+5	≤8.7E+5	≤5.8E+5	≤5.8E+5	3.1E+6	≤5.8E+5	≤5.8E+5	≤5.8E+5
**KB1_17**	≤2.4 E+5	4.7E+4	≤5.0E+5	2.3E+4	2.5E+5	≤3.2E+4	≤5.0E+5	≤3.2E+4	≤3.2E+4	2.4E+6	≤3.2E+4	6.5E+5	≤3.2E+4
**KB1_18**	≤6.0 E+4	4.8E+5	≤1.0E+6	2.1E+5	8.9E+5	≤4.8E+5	6.3E+8	≤4.8E+5	≤4.8E+5	2.3E+6	≤4.8E+5	3.2E+6	≤4.8E+5
**KB1_19**	≤1.7 E+5	2.1E+5	9.1E+05	1.0E+5	2.6E+5	≤6.1E+5	1.3E+8	4.2E+6	1.5E+6	2.9E+6	1.2E+6	1.2E+7	≤6.1E+5
**KB1_23**	≤1.8 E+5	7.0E+4	5.3E+8	3.6E+4	3.3E+5	≤7.3E+5	3.7E+10	2.6E+8	≤7.3E+5	≤7.3E+5	≤7.3E+5	≤7.3E+5	≤7.3E+5
**KB1_25**	≤7.0E+4	3.0E+3	≤8.2E+5	3.0E+3	2.0E+5	≤3.2E+4	≤8.2E+5	≤3.2E+4	1.9E+5	5.8E+6	1.6E+6	3.6E+6	≤8.2E+2
Geo 16S	NA	NA	≤1.1E+6	NA	NA	≤4.1E+4	5.0E+9	7.7E+6	≤4.1E+4	≤4.1E+4	≤4.1E+4	≤4.1E+4	3.0E+5
Geo rdhA	NA	NA	≤4.6E+5	NA	NA	≤2.1E+4	6.6E+9	4.0E+7	≤2.1E+4	1.7E+5	≤2.1E+4	≤2.1E+4	2.1E+6

Dhc = *Dehalococcoides*; inoc. = inoculum; mo. = month; mos. = months; NA = not analyzed; prebiost = prebiostimulation; prebioaug = prebioaugmentation; postbioaug = postbioaugmentation. ≤ MDL

Note 1: The difference between duplicate measurements was usually around 5% or less of the absolute value of each duplicate measurement.

Note 2: Prior to any treatment *Dhc* counts were below the method detection limit in all wells, except in effluent where they were 1E+5 gene copies per L.

Note 3: *Dhc* and *rdhA* counts for SW75 and EFF at month 10 were below the MDL (data not shown in Table above)

Table S9. Detection limits for the quantification via qPCR of the 16S rRNA genes of *Dehalococcoides*, *Geobacter* and the *rdhA* genes for different sample batches (field samples and lab cultures).

	**SABRE**	**ISSO prebiostim**	**ISSO postbiostim and postbioaug**	**KB-1 cultures and inoculum**
	**Gene copies L^{-1}**	**Gene copies L^{-1}**	**Gene copies L^{-1}**	**Gene copies mL^{-1}**
Dhc 16S	6.2+5	7.1E+4	3.1E+3	8.9E+2
**KB1_1**	NA	NA	NA	3.6E+2
**KB1_4**	8.3E+5	2.1E+5	1.4E+4	1.1E+3
**KB1_5**	NA	NA	NA	7.4E+2
**KB1_6**	3.4E+5	4.8E+4	2.2E+3	1.0E+3
**KB1_11**	9.7E+5	2.4E+5	1.5E+4	1.1E+3
**rdhA12**	4.6E+5	1.8E+5	1.1E+4	4.2E+2
**KB1_14**	4.2E+5	6.2E+4	8.1E+3	6.8E+2
**KB1_15**	2.8E+4	6.0E+4	6.4E+3	8.9E+2
**KB1_16**	5.8E+5	1.8E+5	1.2E+4	8.7E+2
**KB1_17**	3.2E+4	2.4E+5	5.5E+3	5.0E+2
**KB1_18**	4.8E+5	6.0E+4	7.2E+3	1.0E+3
**KB1_19**	6.1E+5	1.7E+5	8.8E+3	8.2E+2
**KB1_23**	7.3E+5	1.8E+5	1.5E+4	9.5E+2
**KB1_25**	3.2E+4	7.0E+4	3.0E+3	8.2E+2
**KB1_27**	NA	NA	NA	4.6E+2
Geo 16S	4.1E+4	NA	NA	1.1E+3
**Geo *rdhA***	2.1E+4	NA	NA	4.6E+2

NA = not analyzed; postbioaug = postbioaugmentation; postbiostim = postbiostimulation; prebiostim = prebiostimulation.

Note: Detection limits reflect the extraction volumes used: L for groundwater samples and mL for cultures.

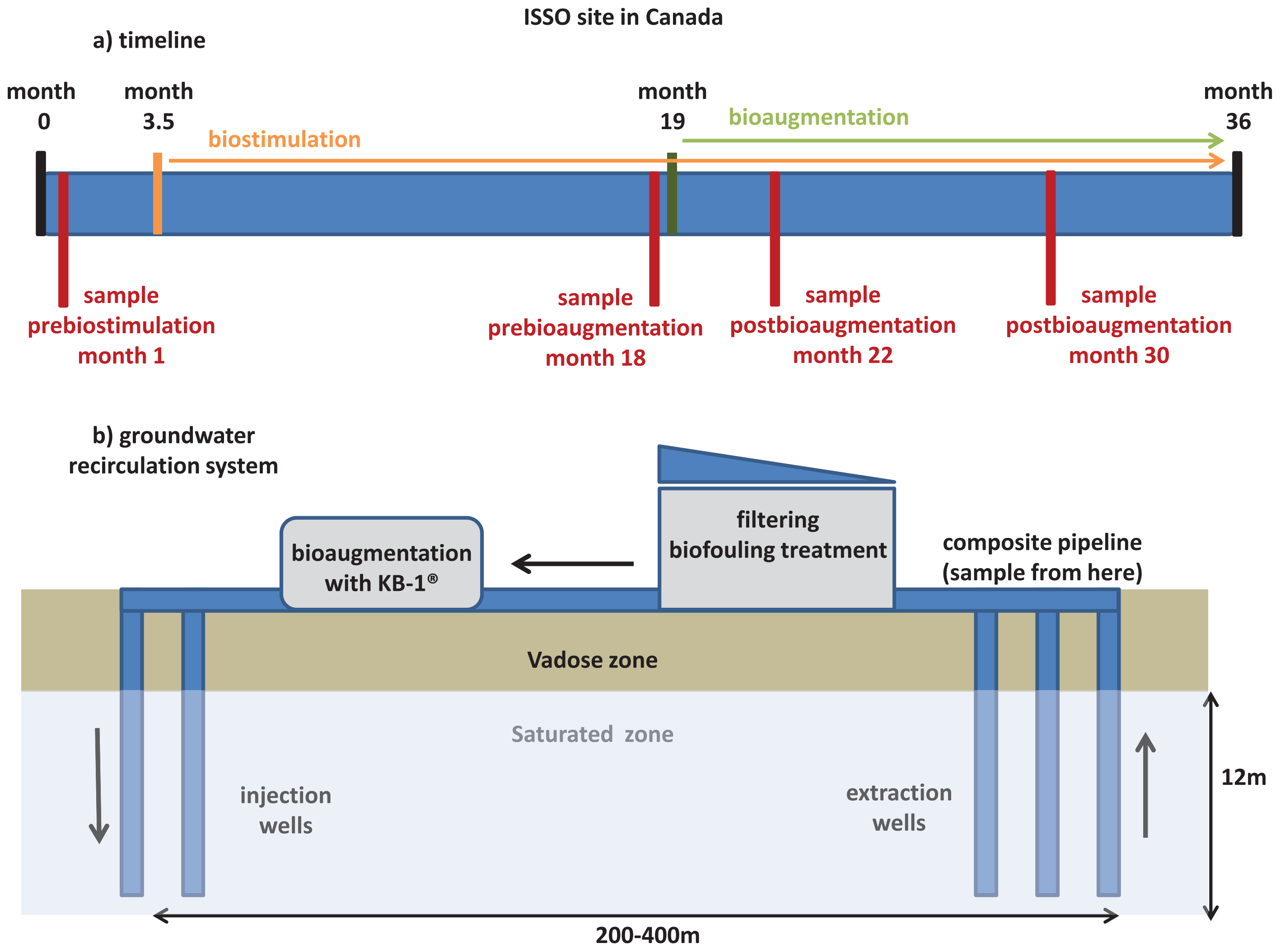

Figure S1. The ISSO site in Canada. a) Timeline including sampling dates and other relevant events such as the start of the biostiumulation and bioaugmentation phases. b) Frontal section of the site depicting the direction of the groundwater within the recirculation system.

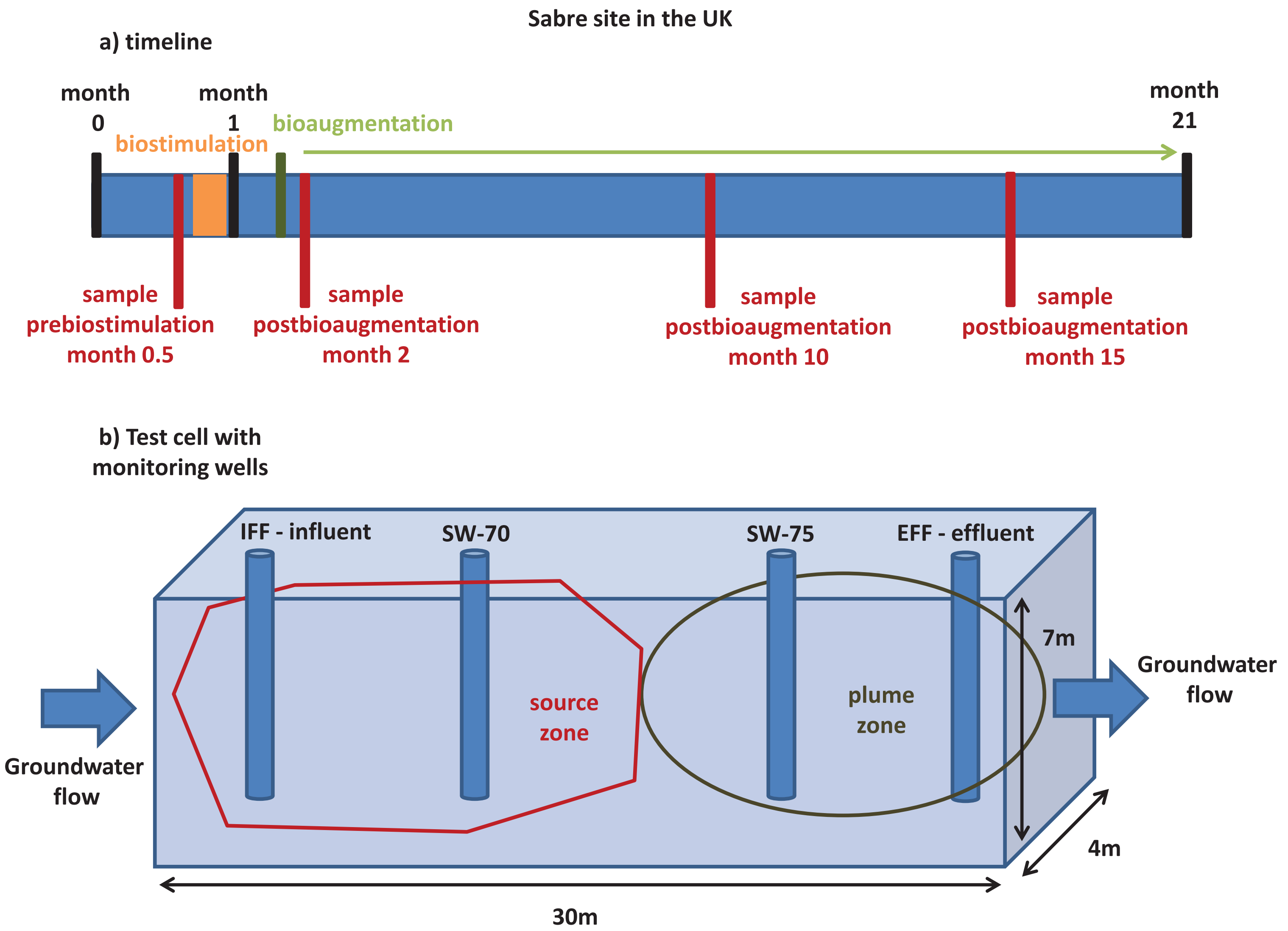

Figure S2. The Sabre site in the UK. a)Timeline including sampling dates and other relevant events such as the start of the biostimulation and bioaugmentation phases. b) Sketch of the pilot test cell with sampling locations including four monitoring wells: influent, SW-70, SW-75 and effluent.

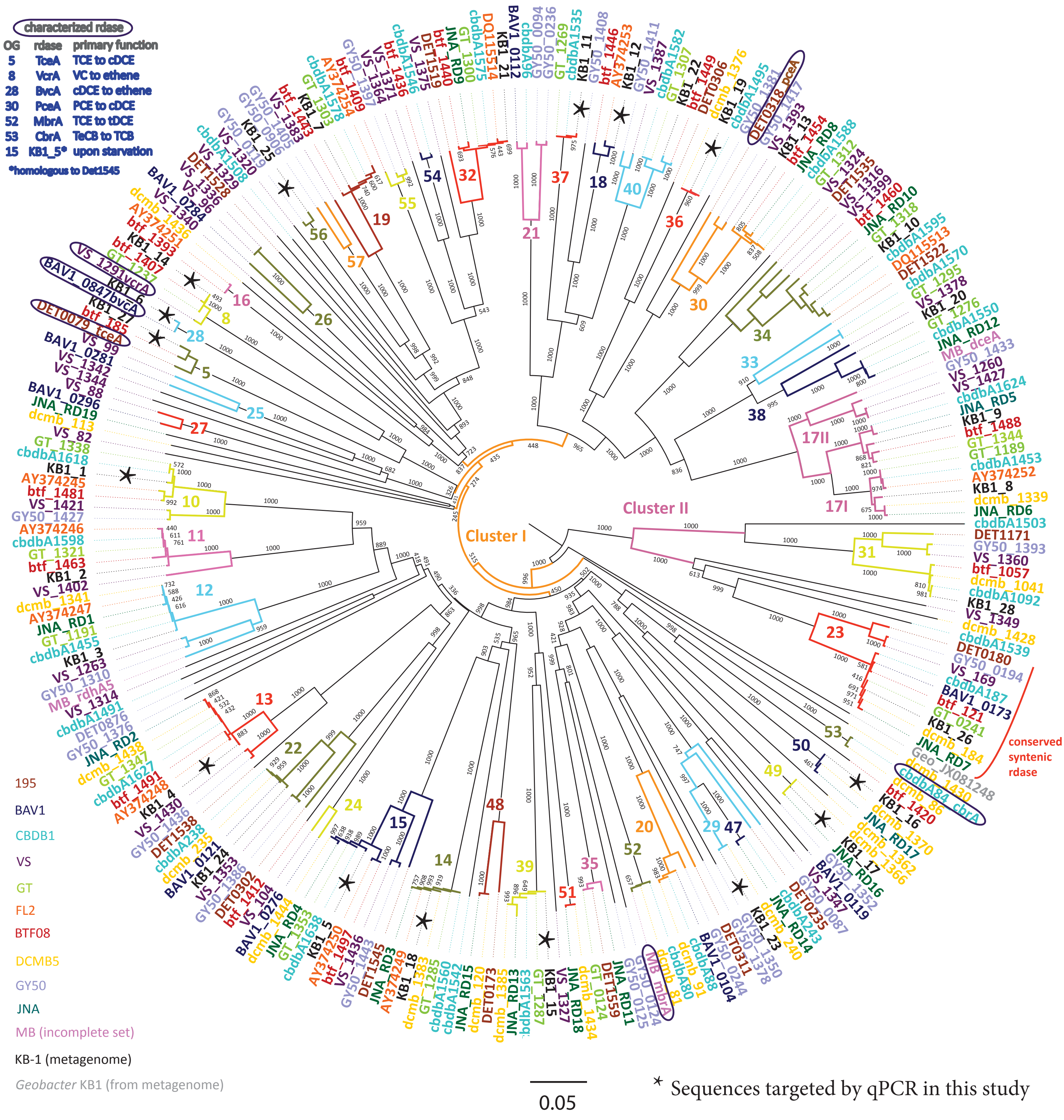

Figure S3. Phylogenetic tree with bootstraps based on the full nucleotide sequences of the *rdhA* genes of eleven isolated *D. mccartyi* strains as well as those found in the mixed dechlorinating culture KB-1, including an *rdhA* gene found in *Geobacter* KB-1. The scale at the bottom of the tree represents the number of susbtitutions per nucleotide. The numbers in colours represent *rdhA* ortholog groups (OGs). Genbank accession numbers for the nucleotide sequences of the MB strain are as follows: EU625401.1 for MB_rdhA5, GU120391.1 for mbrA and EU625402 for dceA1.

References cited in Supporting Information

1. Hug LA, Maphosa F, Leys D, Loffler FE, Smidt H, Edwards EA, Adrian L (2013). Overview of organohalide-respiring bacteria and a proposal for a classification system for reductive dehalogenases. Phil Trans Royal Soc B: Biol Sci, 368(1616): 20120322.

2. Tang S, Chan WW, Fletcher KE, Liang X, Seifert J, Löffler FE, Edwards EA. 2013. Functional characterization of reductive dehalogenases using blue native polyacrylamide gel electrophoresis. Appl Environ Microbiol 79(3):974–981.

3. Duhamel M, Edwards EA. Microbial composition of chlorinated ethene-degrading cultures dominated by *Dehalococcoides*. FEMS Microbiol Ecol 26(58): 538–549.

4. Waller AS, Krajmalnik-Brown R. Löffler FE, Edwards EA. Multiple reductive-dehalogenase-homologous genes are simultaneously transcribed during dechlorination by *Dehalococcoides*-containing cultures. Appl Environ Microbiol 71: 8257–8264.

5. Fung JM, Morris RM, Adrian L, Zinder SH. Expression of reductive dehalogenase genes in *Dehalococcoides ethenogenes* strain 195 growing on tetrachloroethene, trichloroethene, or 2,3-dichlorophenol. Appl Environ Microbiol 73: 4439-45.
